# Increased iron deposition in nucleus accumbens associated with disease progression and chronicity in migraine

**DOI:** 10.1186/s12916-023-02855-1

**Published:** 2023-04-07

**Authors:** Xiaopei Xu, Mengting Zhou, Xiao Wu, Fangling Zhao, Xiao Luo, Kaicheng Li, Qingze Zeng, Jiahui He, Hongrong Cheng, Xiaojun Guan, Peiyu Huang, Minming Zhang, Kaiming Liu

**Affiliations:** 1 Department of Radiology, The Second Affiliated Hospital, Zhejiang University School of Medicine, No 88 Jiefang Road, Hangzhou, Zhejiang China; 2 Department of Neurology, The Second Affiliated Hospital, Zhejiang University School of Medicine, No 88 Jiefang Road, Hangzhou, Zhejiang China

**Keywords:** Migraine, Iron deposition, Nucleus accumbens, Disease burden, Chronicity, Neuroimaging biomarkers

## Abstract

**Background:**

Migraine is one of the world’s most prevalent and disabling diseases. Despite huge advances in neuroimaging research, more valuable neuroimaging markers are still urgently needed to provide important insights into the brain mechanisms that underlie migraine symptoms. We therefore aim to investigate the regional iron deposition in subcortical nuclei of migraineurs as compared to controls and its association with migraine-related pathophysiological assessments.

**Methods:**

A total of 200 migraineurs (56 chronic migraine [CM], 144 episodic migraine [EM]) and 41 matched controls were recruited. All subjects underwent MRI and clinical variables including frequency/duration of migraine, intensity of migraine, 6-item Headache Impact Test (HIT-6), Migraine Disability Assessment (MIDAS), and Pittsburgh Sleep Quality Index (PSQI) were recorded. Quantitative susceptibility mapping was employed to quantify the regional iron content in subcortical regions. Associations between clinical variables and regional iron deposition were studied as well.

**Results:**

Increased iron deposition in the putamen, caudate, and nucleus accumbens (NAC) was observed in migraineurs more than controls. Meanwhile, patients with CM had a significantly higher volume of iron deposits compared to EM in multiple subcortical nuclei, especially in NAC. Volume of iron in NAC can be used to distinguish patients with CM from EM with a sensitivity of 85.45% and specificity of 71.53%. As the most valuable neuroimaging markers in all of the subcortical nuclei, higher iron deposition in NAC was significantly associated with disease progression, and higher HIT-6, MIDAS, and PSQI.

**Conclusions:**

These findings provide evidence that iron deposition in NAC may be a biomarker for migraine chronicity and migraine-related dysfunctions, thus may help to understand the underlying vascular and neural mechanisms of migraine.

**Trial registration:**

ClinicalTrials.gov, number NCT04939922.

## Background

Migraine is a highly prevalent disorder that imposes an enormous socioeconomic burden. While patients with chronic migraine (CM) only account for 1.4–2.2% of the general population globally [1], they usually have lower health-related quality of life and higher levels of disability [2] compared to patients with episodic migraine (EM). Annually, around 3% of the EM patients evolve to CM [3]; however, the rigorous neural mechanism behind the chronicity of migraine remains incompletely understood.

The pathophysiology of migraine involves both vascular and neural mechanisms [4]. Although it is less clear what drives the activation of neuronal pain pathways in a susceptible patient, there is increasing evidence that the pathophysiology of migraine may, in part, be rooted in the dysfunction of subcortical structures [5–7]. During the migraine triggering process, neurons located in the trigeminal subnucleus caudalis (TNC) transmit glutamatergic processes to the thalamus. Subsequently, the thalamus neurons primarily project to the somatosensory cortex, the insula, and the association cortex [5]. TNC neurons also connect to affective/motivational circuits via the nucleus tractus solitarius and parabrachial nucleus, which have diffuse projections to the hypothalamus, thalamic nuclei, amygdala, insula, and frontal cortex. Finally, TNC neurons project directly to output structures involved in pain modulation, such as the hypothalamus and periaqueductal gray (PAG) [5]. Consequently, subcortical regions play an important role in the neuronal pain pathways of migraine. Meanwhile, during migraine attacks, inflammatory vasoactive peptides promote dilatation of the meningeal vessels [8, 9], and the inflammatory response further contributes to the disruption of the blood–brain barrier (BBB) [10]. The alteration of BBB integrity leads to increased iron permeability and deregulation of iron homeostasis [11]. As an electron facilitator serves many brain functions including myelin production and neurotransmitter synthesis [12], iron has received increasing attention in recent years. Iron dysregulation, such as increased iron accumulation, may lead to the continual generation of radical species and toxic free radicals [13], damage dopamine synthesis [14], and eventually, damage to the nervous system. Hence, investigating the iron deposition in subcortical regions of migraineurs could help to advance our understanding of the underlying mechanisms of the disorder and lead to the development of new and more effective treatments.

Using non-invasive techniques such as T2-weighted and T2*-weighted MR imaging, the signal reduction caused by iron provides us with an indirect way to visualize iron content. In migraineurs, increased iron deposition has been found in the PAG [15–17], putamen, and globus pallidus [16], and an inverse relationship was established between recurrent attacks and iron accumulation. However, previous studies have only focused on limited subcortical brain regions, and other studies [18–20] have supported the different roles of the amygdala, nucleus accumbens, and thalamus in migraine pathophysiological mechanisms, suggesting that these regions deserve equal attention. Furthermore, quantitative analysis of iron deposition has not been performed in a larger population. In this sense, a more comprehensive investigation may contribute to the potential modifiable role of iron accumulation in migraine with functional disability.

With the recent development, quantitative susceptibility mapping (QSM) is a novel post-processing technique to quantitatively assess the magnetic susceptibility of the tissue thus may provide improved image quality for the visualization of the subcortical nucleus [21]. Compared to conventional T2* relaxometry, QSM derives values sensitive to the levels of iron, thus is more selective for iron. Previous studies [22, 23] using QSM showed increased iron deposition in total cerebral gray matter and in cortical regions like precuneus, insula, supramarginal gyrus, and postcentral gyrus in CM. However, cortical susceptibility is more prone to the surface and streaking artifacts appear in the vicinity of large susceptibility gradients [24]. Although these artifacts could partly be suppressed by post-processing methods [25, 26], this makes the subcortical assessments more feasible and readily available in daily practice. Furthermore, the regional iron deposition in subcortical nuclei was not fully investigated thus far. Therefore, in the current study, a susceptibility analysis would provide more valuable information for understanding the neural mechanism of CM.

This study aims to use the QSM to comprehensively investigate the brain iron concentration of subcortical brain nuclei in patients with CM and EM as compared to healthy controls. The relationships between iron deposition and disease course as well as functional disabilities were also investigated.

## Methods

### Participants

This study was approved by the local Institutional Review Board, and written informed consents were obtained from all participants. From September 2021 to January 2023, individuals diagnosed with EM or CM according to the International Classification of Headache Disorders, 3rd edition criteria were selected.

Patients were recruited based on the following inclusion criteria: (1) age: 18–70 years; (2) confirmed diagnosis is EM or CM; (3) history of migraine greater than 1 year. Subjects were excluded if they were (1) high blood pressure; (2) coronary disease; (3) diabetes mellitus; (4) hypercholesterolemia; (5) infectious diseases; (6) chronic inflammatory conditions and other autoimmune conditions; (7) severe systemic diseases; (8) pregnancy or lactation; (9) obesity (body mass index > 30 kg/m^2^); (10) smoking habit; and (11) recent consumption of antiplatelet drugs or vasoactive drugs (> 4 times the medium half-life of the active substance). Age- and sex-matched healthy controls were recruited from the community if they fulfilled all inclusion and exclusion criteria and were free of any headache or psychiatric disorder. Eventually, a total of 200 migraineurs (56 CM, 144 EM) and 41 matched controls were recruited.

### Clinical assessment

All subjects underwent a medical interview including demographic data (age, sex) and personal family histories. For migraineurs, disease duration (measured in years from first symptoms), frequency of migraine attacks per month, migraine days per month, and peak headache pain intensity (measured by visual analog scale (VAS) were registered. The 6-item Headache Impact Test (HIT-6) and Migraine Disability Assessment (MIDAS) were performed to measure the degree of migraine-related functional disability, and Pittsburgh Sleep Quality Index (PSQI) was also performed to assess the sleep quality of migraineurs over the past month.

### Image Acquisition

All the MR images were acquired using a United Imaging MR790 3.0 T scanner (Shanghai, China). T1 weighted images were acquired with a 3D fast spoiled gradient-echo sequence; the parameters were: TR = 6.9 ms, TE = 2.9 ms, flip angle = 9°, inversion time = 1000 ms, field of view = 256 × 240 mm, voxel size = 1 × 1 × 1 mm^3^, 208 sagittal slices. T2 weighted images were acquired with a MATRIX (modulated flip angle technique in refocused imaging with extended echo train, equivalent to CUBE for GE, SPACE for Siemens, and VISTA for Philips) sequence; the parameters were: TR = 3000 ms, TE = 405.46 ms, echo train length = 180, field of view = 256 × 240 mm, voxel size = 0.8 × 0.8 × 0.8 mm^3^, 208 sagittal slices. The susceptibility weighted imaging (SWI) sequence was acquired using a multi-echo 3D gradient echo sequence (GRE) with the following parameters: TR = 36.5 ms, first TE = 3.1 ms, last TE = 30.4 ms, number of echo = 8, bandwidth = 350 kHz, flip angle = 15°, field of view (FOV) = 240 × 240 mm, voxel size = 0.75 × 0.75 × 2 mm^3^.

### Image processing

The QSM images were reconstructed from GRE data using the SEPIA (SuscEptibility mapping PIpeline tool for phAse images) toolbox [27] in the MATLAB program (The Mathworks Inc., Natick MA). Brain extraction was performed on whole-brain magnitude data based on the BET tool in Functional Magnetic Resonance Imaging of the Brain (FMRIB) Software Library v6.0 (FSL; Oxford University, UK; https://fsl.fmrib.ox.ac.uk/fsl/fslwiki/FSL) package from the MEDI toolbox. The phase images were unwrapped with SEGUE [28]. After unwrapping, the background field was removed with the regularisation-enabled SHARP (RESHARP) [29] filtering method. Lastly, magnetic susceptibility was quantitatively calculated using MEDI [29–31], and QSM images were generated. For MEDI, the mean susceptibility value of the cerebrospinal fluid (CSF) within the manually drawn ROI in the posterior lateral ventricles of each subject was used as the susceptibility reference [31, 32]. While there has been no consensus on the determination of a susceptibility reference, most studies used the mean susceptibility value of white matter areas (frontal, occipital, etc.), CSF, or the whole brain as the reference [33]. Nevertheless, a high degree of consensus was demonstrated between the regional susceptibility values using different references (whole brain vs. CSF) by a recent study, and the findings remain repeatable regardless of the choice of different references [34].

**Fig. 1:**
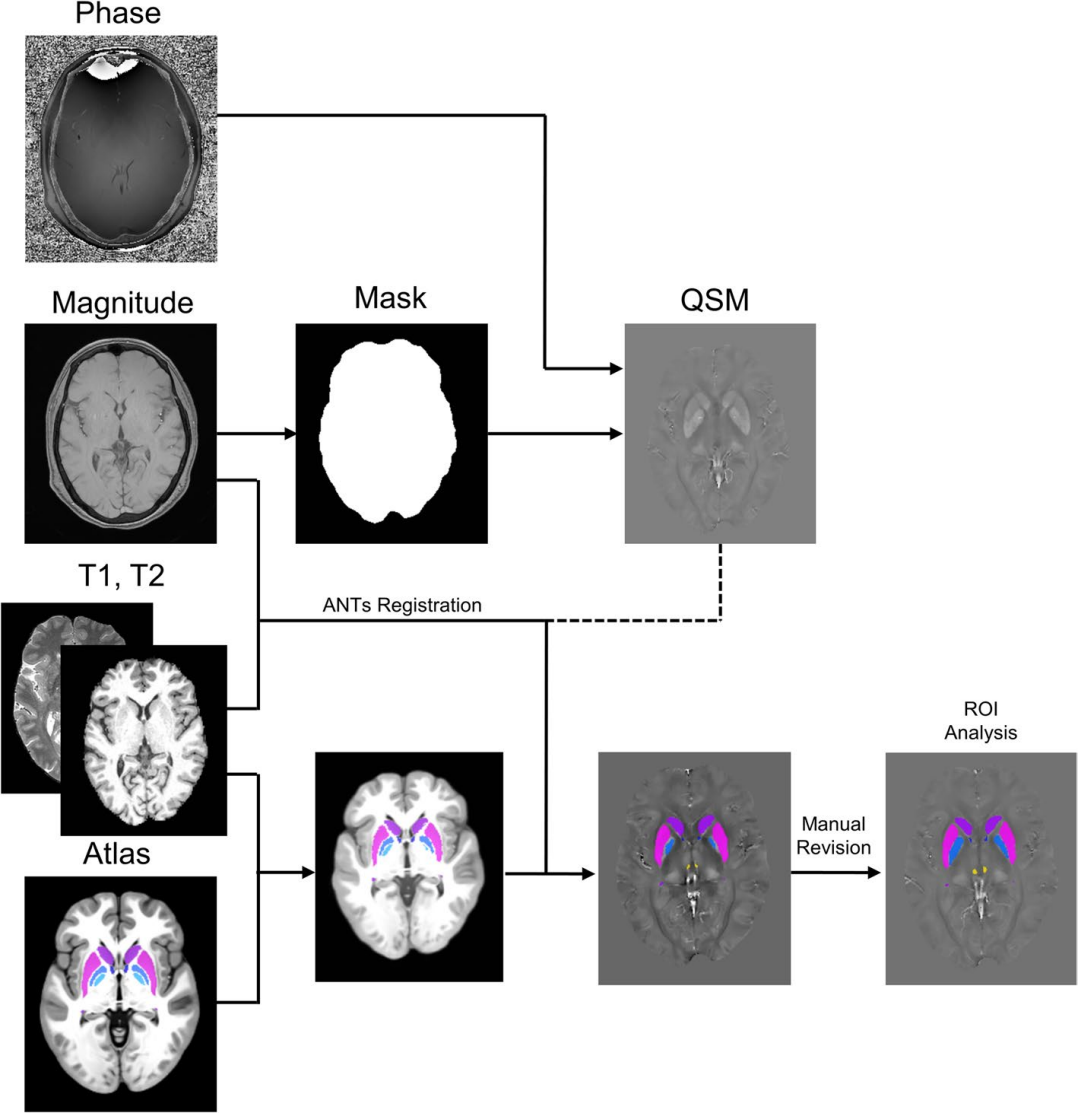
Summarized steps of the pipeline for image preprocessing. The phase images were unwrapped, and the background field was removed with the regularisation-enabled SHARP filtering method. Magnetic susceptibility was quantitatively calculated using MEDI and quantitative susceptibility map (QSM) images were generated. T1, T2, and magnitude images were skull-stripped. By using Advanced Normalization Tool (ANTs), SyN multimodal warping was performed using joint T1 and T2 cost function to transform the high-resolution probabilistic subcortical brain nuclei atlas in CIT168 space to individual space. Eventually, the QSM value of each subcortical nuclei was extracted from each manually refined region of interest (ROI) based on the subcortical nuclei atlas

The magnitude image, in the same space as the QSM image, was used for registration and was obtained by summing the squares of magnitude images among different TEs. 3D T1, T2, and magnitude images were skull-stripped using BET in FSL. By using citatlaskit (https://github.com/jmtyszka/citatlaskit) tool based on Advanced Normalization Tools v2.1 (ANTs; http://stnava.github.io/ANTs/), SyN multimodal warping was performed using joint T1 and T2 cost function to transform the high-resolution probabilistic subcortical brain nuclei atlas in CIT168 space to individual space. The subcortical nuclei atlas in native space was manually refined by a neuroradiologist (X.Xu) with 4 years’ experience to ensure the segmentation precision. Eventually, the mean QSM value of regions of interest (ROI) in native subcortical nuclei based on the human subcortical brain nuclei atlas [35] was extracted to quantify the tissue susceptibility, indicating iron deposition in certain subcortical nucleus (Fig. 1). The high-resolution probabilistic atlas of human subcortical brain nuclei contains the following brain regions: putamen (Pu), caudate (Ca), nucleus Acumbens (NAC), extended amygdala (EXA), external globus pallidus (GPe), internal globus pallidus (GPi), ventral pallidum (VeP), substantianigra pars compacta (SNc), substantia nigra pars reticulata (SNr), parabrachial pigmented nucleus (PBP), subthalamic nucleus (STH), ventral tegmentum (VTA), hypothalamus (HTH), red nucleus (RN), mammillary nucleus (MN), habenular nuclei (HN).

### Statistical analysis

Sex was recorded as binary variables. Age, disease duration, migraine attacks per month, disease duration, migraine days per month, VAS, HIT-6, MIDAS, and PSQI were recorded as continuous variables, and one-sample Kolmogorov–Smirnov test was used to check the normality of all continuous variables. Demographics and clinical variable were compared between controls and migraineurs, and between CM and EM using independent samples *t*-test and Mann–Whitney test for continuous variables, and the chi-squared test for proportions.

Analysis of variance (ANOVA) was performed to evaluate the regional difference in iron-related metrics among the three groups. Subsequently, Bonferroni post hoc analysis was applied to analyze the difference between each of the two groups. Partial correlation analysis was conducted to detect the potential relationship between regional iron-related metric and clinical variables in migraine patients, and in patients with CM and EM, respectively. All analyses were adjusted for age and sex. Bonferroni correction for the problem of multiple comparisons in multiple-region level, and to further control for the type I error was performed. A significance level of *p* < 0.05 was set for all statistical tests. Receiver operating characteristics (ROC) curve was applied to evaluate the diagnostic efficacy of the QSM value, and area under the curve (AUC) was recognized as reasonable diagnostic valuable with AUC > 0.7. SPSS 22.0 (SPSS, Chicago, IL) was used for all the statistical analyses mentioned above.

## Results

### Demographics

A total of 200 patients with migraine as well as 41 normal controls (both age and sex matched) were recruited, and all of them underwent the MRI scan. For patients with migraine, 144 of them were episodic, while the rest 56 patients were chronic. Patient demographics and statistical significance of group comparisons are summarized in Table 1. There was no significant difference in age or gender between migraineurs and normal controls. Mean age of patients with CM was 37.9 ± 11.9 years, and 75.4% were female. Mean age of patients with EM was 47.5 ± 15.4 years, and 77.8% were female. There was a statistically significant difference in age between patients with CM and those with EM (*p* < 0.001). Patients with CM showed significantly longer disease duration (*p* < 0.01), higher frequency of attacks (*p* < 0.01), more migraine days per month (*p* < 0.01) when compared to EM. A higher VAS (*p* < 0.01), HIT-6 (*p* < 0.01), MIDAS (*p* < 0.01), and PSQI (*p* < 0.01) could also be observed in patients with CM. Moreover, higher PSQI was associated with higher VAS (*r* = 0.225, *p* = 0.004), HIT-6 (*r* = 0.741, *p* < 0.001), and MIDAS (*r* = 0.764,* p* < 0.001).

**Table 1:** Comparisons of demographic and clinical data between migraineurs and controls

	Migraineurs (*n* = 200)		Control (*n* = 41)	*p* value
	Episodic (*n* = 144)	Chronic (*n* = 56)		
Age, years	40.6 ± 12.3		43.9 ± 12.3	0.120
Male, *n* (%)	46 (23.0%)		14 (34.1%)	0.132
Disease duration, years	10.2 ± 8.7	18.5 ± 11.0	-	< 0.001
Monthly migraine attacks	3.0 ± 2.5	15.4 ± 9.0	-	< 0.001
Monthly migraine days	3.4 ± 3.0	21.0 ± 6.2	-	< 0.001
Peak headache pain intensity (VAS)	6.4 ± 1.1	6.9 ± 0.9	-	< 0.05
HIT-6	49.8 ± 9.9	66.1 ± 8.1	-	< 0.001
MIDAS	6.8 ± 8.1	16.8 ± 8.9	-	< 0.001
PSQI	3.6 ± 2.6	13.3 ± 4.0	-	< 0.001
HADS-D	3.5 ± 2.5	5.2 ± 2.4	-	< 0.001
HADS-A	3.4 ± 2.6	5.4 ± 3.0	-	< 0.001
Analgesics, *n* (%)	68 (47.2%)	33 (58.9%)	-	0.137
Preventive medication, *n* (%)	42 (29.2%)	27 (48.2%)	-	< 0.05
Antidepressant, *n* (%)	9 (6.3%)	4 (7.1%)	-	0.818
Antiepileptic, *n* (%)	23 (16.0%)	12 (21.4%)	-	0.362
Beta-blocker, *n* (%)	12 (8.3%)	4 (7.1%)	-	0.781
CGRP pathway, *n* (%)	0	0	-	/
Lisinopril or candesartan, *n* (%)	2 (1.4%)	0	-	0.375
Onabotulinumtoxin A, *n* (%)	0	3(5.4%)	-	< 0.05
Other, *n* (%)	21 (14.6%)	8 (14.3%)		0.957

*VAS* Visual analog scale, *HIT-6* The 6-item Headache Impact Test, *MIDAS* Migraine; Disability Assessment, *PSQI* Pittsburgh Sleep Quality Index, *HADS* Hospital Anxiety and Depression Scale, *HADS-D* HADS Depression, *HADS-A* HADS Anxiety

### Regional comparisons of iron-related metric between groups

**Fig. 2:**
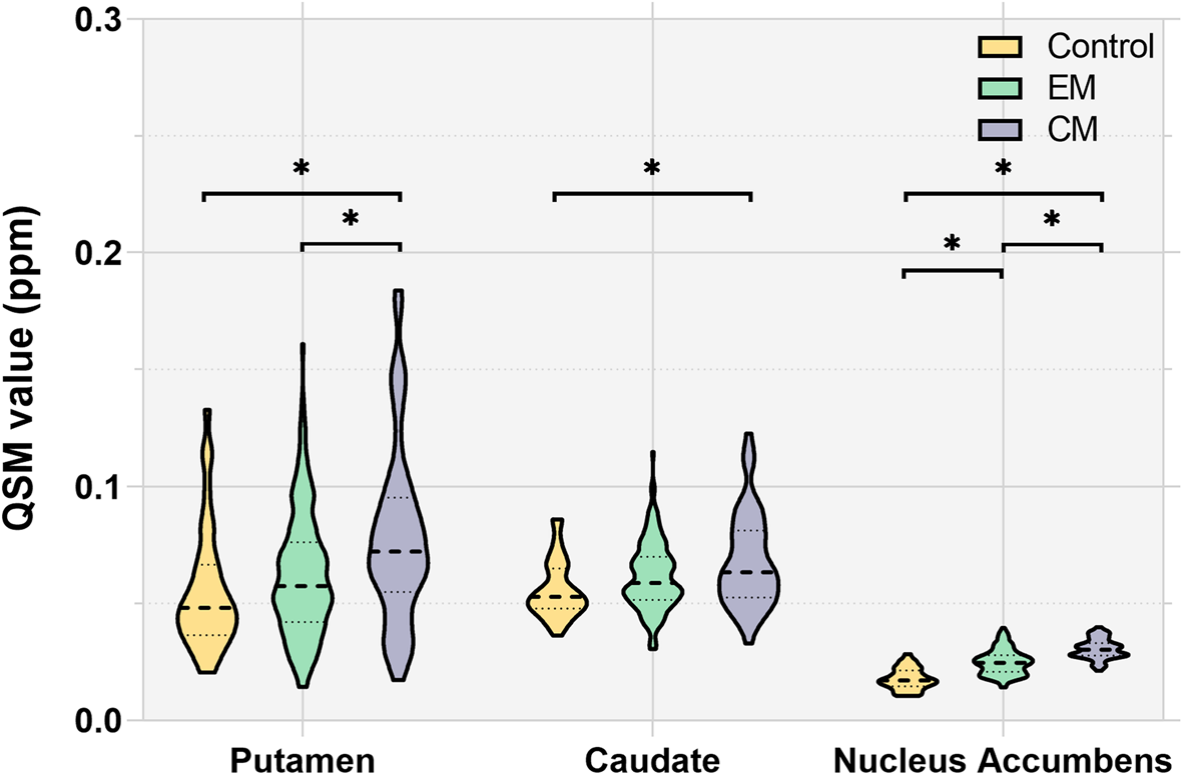
Iron content (measured by QSM value) distributions in the putamen, caudate, and nucleus accumbens among three groups. * indicates the significant difference (*p* < 0.05) of regional iron deposits between groups. EM, episodic migraine; CM, chronic migraine

Significantly higher QSM value was observed in Pu (*p* = 0.001), Ca (*p* = 0.002), and NAC (*p* < 0.001) in patients with CM compared to controls (Fig. 2). Patients with EM showed significantly higher QSM values in NAC (*p* < 0.001) compared to controls. When compared to patients with EM, patients with CM had a significantly higher QSM value in Pu (*p* = 0.002), NAC (*p* < 0.001), SNc (*p* = 0.018), PBP (*p* = 0.003), and HN (*p* = 0.017). The difference between migraineurs and controls was not significant in other subcortical brain nuclei.

### ROC analysis of the QSM value

**Fig. 3:**
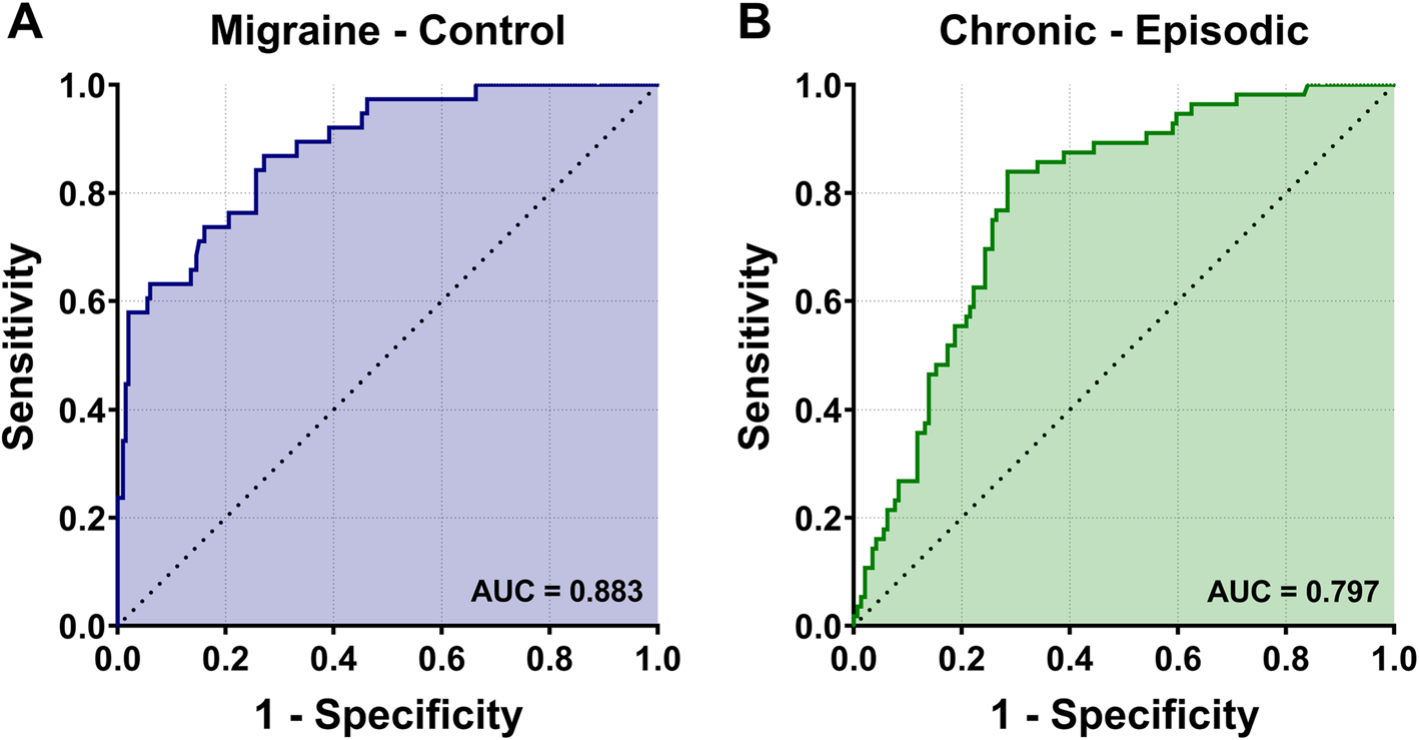
Receiver operating characteristic (ROC) curves for iron deposits in nucleus accumbens (NAC). **A** ROC curve for the QSM value of NAC to diagnose migraineurs from controls. The area under the curve (AUC) was 0.883. **B** ROC curve for the QSM value of NAC to diagnose patients with chronic migraine from those with episodic migraine. The AUC was 0.797

After calculation of receiver operating characteristic curves (Fig. 3), the area under curve (AUC) for migraineurs regarding QSM value in NAC was 0.883 (95% CI 0.826–0.939). The optimal threshold was 22.91 ppb, which would identify 72.9% of patients with migraine (sensitivity) and 86.8% of patients without (specificity). Similarly, the AUC for the QSM value of NAC was 0.797 (95% CI 0.734–0.860), and the optimal cut-off value was set as 27.23 ppb with the sensitivity 85.45% and specificity 71.53% in distinguishing CM from EM.

### Relationship between iron-related metric and clinical variables

**Fig. 4:**
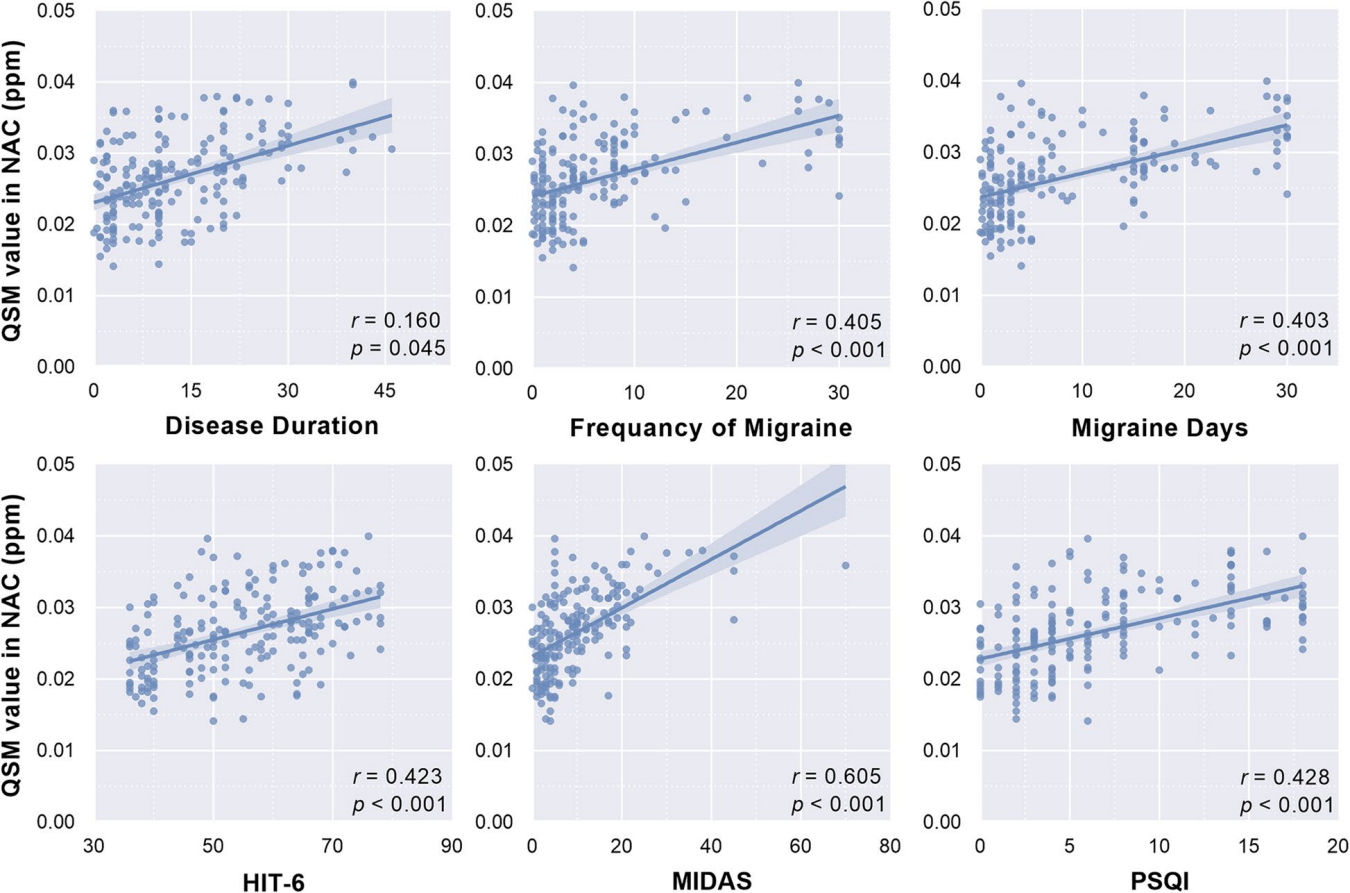
Correlation between iron deposits in nucleus accumbens (NAC) and clinical variables in migraineurs. HIT-6, The 6-item Headache Impact Test; MIDAS, Migraine Disability Assessment; PSQI, Pittsburgh Sleep Quality Index

In migraineurs, the QSM values of NAC were significantly associated with longer disease duration (*r* = 0.160, *p* = 0.045), higher frequency of attacks (*r* = 0.405, *p* < 0.001), more migraine days per month (*r* = 0.403, *p* < 0.001), and higher scores in HIT-6 (*r* = 0.423, *p* < 0.001), MIDAS (*r* = 0.605, *p* < 0.001), and PSQI (*r* = 0.428, *p* < 0.001) as shown in Fig. 4. For each patient group, the QSM values of NAC in CM were significantly associated with higher frequency of attacks (*r* = 0.581,* p* = 0.006), more migraine days per month (*r* = 0.528, *p* = 0.014) and higher scores in MIDAS (*r* = 0.650, *p* = 0.001). In patients with EM, the QSM values of NAC were significantly associated with higher scores in MIDAS (*r* = 0.515, *p* = 0.005) and PSQI (*r* = 0.403, *p* = 0.033). Moreover, the QSM value of VeP was negatively correlated with frequency of attacks (*r* =  − 0.207, *p* = 0.009), migraine days per month (*r* =  − 0.246, *p* = 0.002), HIT-6 (*r* =  − 0.182, *p* = 0.022), and PSQI (*r* =  − 0.241, *p* = 0.002). Higher HIT-6 (*r* = 0.160, *p* = 0.044) and MIDAS (*r* = 0.182, *p* = 0.022) were associated with higher QSM value in HN.

## Discussion

Our study demonstrated that migraineurs had increased iron deposition in Pu, Ca, and NAC than healthy controls. Meanwhile, patients with CM had a significantly higher volume of iron deposits in multiple subcortical brain nuclei including Pu, Ca, NAC, SNc, PBP, and HN compared to EM. Volume of iron in NAC can be used to distinguish patients with migraine from controls with a sensitivity of 72.9% and specificity of 86.8, and CM from EM with a sensitivity of 85.45% and specificity of 71.53%. Moreover, greater iron deposition in NAC was significantly associated with greater migraine burden, as measured by longer disease duration, higher frequency of attacks, more migraine days per month, and higher scores in HIT-6, MIDAS, and PSQI.

Although increased iron deposition of subcortical nuclei has been reported in migraine patients, there is a lack of comprehensive subcortical nucleus and systematic comparison. Welch et al. [36] found increased iron accumulation in PAG in patients with chronic daily headaches, suggesting a selectively impaired iron homeostasis in migraineurs, possibly caused by repeated migraine attacks. Another study [37] later confirmed these findings and showed increased iron concentration in Pu, RN, and GP in the younger migraineurs compared to controls. Moulton et al. [38] reported altered functional connectivity in the basal ganglia notably the Pu and Ca compared to normal controls, accentuated by frequency of migraine attacks in migraine patients. Our study showed increased iron-related metrics, as measured by increased QSM value, in Pu, Ca, and NAC in migraineurs when compared to healthy controls. We found that patients with CM had higher iron accumulation in Pu, Ca, NAC, SNc, PBP, and HN than EM. The increased iron levels in the brain, especially in subcortical regions around the basal ganglia, might be related to the abnormal metabolical activities in specific regions, and a potentially higher vulnerability to iron-induced oxidative stress [39].

Repetitive episodes of neuroinflammation and hyperoxia lead to iron redistribution and iron unbalance in migraine patients [40, 41], which could result in an increase in BBB permeability [42] and allow the release of inflammatory mediators, free radicals, vascular endothelial growth factor, matrix metalloproteinases and microRNAs [43]. Increased iron loads and iron-mediated free radical production further caused degeneration of endothelial cells and opening of the BBB [44], thus resembling a vicious circle. Eventually, an excessive amount of iron deposits render the brain more vulnerable to oxidative stress, and thus may cause basal ganglia dysfunction by damaging synapses or modulating protein synthesis, leading to altered local levels of neurotransmitters [45]. Considering the significant role basal ganglia plays in the pathophysiology of pain in migraine [46–48], our study provides further evidence that structural, metabolic, and functional alteration in subcortical nuclei might associate with increased migraine burden and disease-related disability during repeated episodes of migraine. Considering we found larger iron deposits in a patient with CM than those with EM, subcortical nuclei like Pu, Ca, NAC, and SN could be related to migraine chronicity.

Our correlation analysis showed that greater iron deposition in NAC was significantly associated with greater migraine burden, as measured by longer disease duration, higher frequency of attacks, and more migraine days per month, suggesting a relationship between recurring attacks and accumulation of iron [15, 16, 36]. Higher concentration of transferrin receptors in NAC, high iron content in glial cells, and impaired iron homeostasis are possibly associated with neuronal dysfunction or neuronal damage in repeatedly activated networks involved in nociception. A previous study [17] hypothesized that repeated migraine attacks could increase free radical cell damage and thus may lead to increased iron deposition could contribute to migraine chronicity. Stronger functional connectivity of NAC to medial prefrontal cortex (mPFC-NAC) was also found in patients with chronic pain and was positively correlated with pain chronicity [49, 50]. Liu et al. [20] observed significantly decreased regional CBF value in left NAC in CM compared to controls, which might reflect a compensatory mechanism as activation of the mPFC-NAC pathway. Considering that decreased CBF is linked with BBB compromise, and a compensatory increase in CBV may lead to reperfusion injury on BBB [51], the increased iron accumulation in NAC might result from the BBB leakage caused by focal hypoperfusion. Eventually, the QSM value of NAC was identified in the current study to distinguish patients with CM from EM with a sensitivity of 85.45% and specificity of 71.53% (AUC = 0.797). Our study thus provides further evidence for the application of QSM in daily clinical practice to discriminate CM patients.

If increased iron concentrations in NAC would play a role in the migraine chronicity, this might theoretically reflect a defective central pain processing system related to dysfunctions in several domains. NAC is located at the junction of the basal nucleus and the marginal system. The outer part of the septum and the inner and lower part of the caudate nucleus are connected to the pre-olfactory nucleus, and the ventral side is the ventral pale sphere and olfactory nodules. NAC plays important role in reward and punishment mechanisms, but studies on NAC and migraine are limited. The current study found that increased iron deposits in NAC were also associated with a higher level of migraine-related functional disability, as measured by HIT-6 and MIDAS, which are both widely accepted measures to assess headache-related disability and its impact on quality of life. As a key node of neural circuits projecting to multiple pain structures and mediating motivated behaviors [52, 53], NAC is associated with pain medication and plays a role in migraine and hyperalgesia comorbidities when functionally disrupted. A study shows that NAC and pain sensitization are closely related to chronic pain, neurogenesis of medium spiny neurons in the NAC continues into adulthood and is enhanced by pathological pain [54]. Besides, there are also studies [55] that provide evidence that lower NAC volume confers risk for developing chronic pain, and altered NAC activity is a signature of the state of chronic pain. These evidence emphasize the potential role of NAC as a target brain region to track patient disability and aid in the monitoring of treatment regime.

In addition, increased regional iron deposits of NAC were associated with worse sleep quality in migraineurs. Migraineurs usually have worse sleep quality than non-migraineurs [56–58], and our study showed that such a condition is even worse in patients with CM than EM. This association between migraine and sleep disorders is underlined by the intimate relationship in the clinical presentation [57, 59, 60] and by the presence of shared anatomical pathways [61]. During sleep, the BBB and fluid systems play essential roles in the removal of metabolic overload. While sleep can promote toxic metabolic clearance [62], sleep disruption may result in the accumulation of neurotoxic waste products. In patients with primary insomnia [63], significantly increased iron deposition in multiple subcortical nuclei was observed, indicating the important role of iron concentration as a biomarker for sleep disorders. Meanwhile, NAC is a new regulating area for sleep through the integration of motivational stimuli [64]. This might explain why NAC is particularly prone to focal iron deposition in migraineurs.

### Limitations

Despite the novelty of the current study, this prospective study is still prone to several limitations. One important limitation is the fact that our results are based on cross-sectional observation, longitudinal data are needed to justify a such conclusion. Second, the current study included patients across a wide age range. While this approach allowed us to observe for a diverse sample of migraineurs regardless of age, it also limited our ability to draw conclusions about age-specific effects and their associated comorbidities. For instance, elderly patients are usually more prone to depression [65]. Considering patients with significant clinical depression might influence the results, we have excluded subjects who scored 11 or higher on HADS for patients referred to our headache clinic. Future studies focusing on the specific hypotheses about different age groups and their clinical characteristics for migraine and associated psychological problems might help us to understand the problem. Third, the patients with CM are significantly older than patients with EM. In the current study, age is positively associated with widespread iron deposition in subcortical nuclei, which is consistent with the known age-related iron deposition in both cortical and subcortical regions [66–69] despite the high spatial variation in iron distribution. Gender has also been associated with the iron deposition. Our study showed a lower level of iron concentration in multiple subcortical nuclei of women and is in agreement with previous studies [70, 71]. To control for the effect of age and gender on iron concentrations, we have regressed out age and sex as covariates of no interest. Model adjustment alone could not completely eliminate the effect of age and sex, thus future studies are warranted to better address this issue. Finally, while the structural T1 images were collected, the analysis of subcortical structural changes was not included in the current study. Future studies to explore the structural changes of the brain and their relationship with iron deposition in patients with migraine would further our understanding of the spatiotemporal patterns of iron-related neurodegeneration.

## Conclusions

In conclusion, we have successfully demonstrated that there is an increased iron deposition in multiple subcortical nuclei, especially NAC, in patients with CM, and the regional iron accumulation level in NAC could be used to distinguish CM patients from EM. More importantly, the increased iron deposition in NAC was associated with higher disease burden, higher migraine-related disability, and worse sleep quality, suggesting a potential role as a neuroimaging marker to track patient disability and aid in the monitoring of treatment regime. These results provided further evidence for future research efforts to understand the underlying vascular and neural mechanisms behind the pathophysiology of migraine.

## Data Availability

The data that support the findings of this study are not publicly available due to privacy or ethical restrictions. Data are however available from the corresponding authors upon reasonable request and with permission after the completion of the study.

## Abbreviations

AUC: Area under the curve
BBB: Blood-brain barrier
Ca: Caudate
CAMERA: Cerebral Abnormalities in Migraine, an Epidemiological Risk Analysis
CM: Chronic migraine
EM: Episodic migraine
EXA: Extended amygdala
FMRIB: Functional Magnetic Resonance Imaging of the Brain
FOV: Field of view
GPe: External globus pallidus
GPi: Internal globus pallidus
GRE: Gradient echo sequence
HIT-6: 6-Item Headache Impact Test
HN: Habenular nuclei
HTH: Hypothalamus
ICDH-III: International Classification of Headache Disorders, 3rd edition
MATRIX: Modulated flip angle technique in refocused imaging with extended echo train
MIDAS: Migraine Disability Assessment
MN: Mammillary nucleus
mPFC-NAC: NAC to medial prefrontal cortex
NAC: Nucleus accumbens
PBP: Parabrachial pigmented nucleus
PSQI: Pittsburgh Sleep Quality Index
Pu: Putamen
QSM: Quantitative susceptibility mapping
RESHARP: Regularisation-enabled SHARP
RN: Red nucleus
ROC: Receiver operating characteristics
ROI: Regions of interest
SEPIA: SuscEptibility mapping PIpeline tool for phAse images
SNc: Substantianigra pars compacta
SNr: Substantia nigra pars reticulata
STH: Subthalamic nucleus
SWI: Susceptibility weighted imaging
VAS: Visual analog scale
VeP: Ventral pallidum
VTA: Ventral tegmentum
